# ‘Jones hybrid’ hickory: a case study in *Carya* curation

**DOI:** 10.1186/s40064-016-3531-4

**Published:** 2016-10-24

**Authors:** L. J. Grauke, M. Azucena Mendoza-Herrera, David M. Stelly, Patricia E. Klein

**Affiliations:** 1 National Collection of Genetic Resources for Pecans and Hickories, USDA ARS Pecan Breeding and Genetics, 10200 FM 50, Somerville, TX USA; 2 Department of Entomology (formerly Post-Doc, Department of Horticultural Sciences), Texas A&M University, College Station, TX 77843 USA; 3 Department of Horticultural Sciences, Texas A&M University, College Station, TX 77843 USA; 4 Department of Soil and Crop Sciences, Texas A&M University, 370 Olsen Blvd., College Station, TX 77843-2474 USA; 5 Department of Horticultural Sciences, Texas A&M University, 154A Borlaug Center, College Station, TX 77843 USA

**Keywords:** *Carya ovata*, *Carya cordiformis*, *Carya* × *laneyi*, *Carya illinoinensis*, Microsatellite profiles, Morphology

## Abstract

‘Jones Hybrid’ hickory is an accession in the National Collection of Genetic Resources for Pecans and Hickories for which information about origin, identity and characteristics is very incomplete. Phenotypic and genetic profiles, when examined in the context of historic literature, provide evidence that the accession in question is ‘Siers’ a cultivar of *Carya* × *laneyi* (an interspecific hybrid between *C. ovata* and *C. cordiformis*). The accession has traits that make it interesting in the pecan breeding program, with potential for both rootstock and scion development. The tall, slender tree form of ‘Jones Hybrid’ is a trait that could be valuable in commercial pecan cultivars, allowing increased tree densities and reducing the need for expensive hedging operations. Tree size reduction is a goal to be pursued in scion selection and rootstock development, with each goal requiring assessment of reproductive potential of the accession.

## Background

The National Collection of Genetic Resources for Pecans and Hickories (NCGR *Carya*) is the name given to what was formerly the National Clonal Germplasm Repository for Pecans and Hickories, designated as part of the National Plant Germplasm System in 1984 (Postman et al. 2006). The foundation of the NCGR *Carya* was grafted trees of pecan [*Carya illinoinensis* (Wangenh.) K. Koch] amassed from 1933 until his retirement in 1968 by Louis D Romberg, the first pecan breeder in the United States Department of Agriculture, for use in breeding pecans. Other staff members at what was then the USDA Pecan Field Station in Brownwood, Texas, added to the collection by grafting pecans and other hickories. The name change from “Clonal Germplasm Repository” to “Collection of Genetic Resources” more accurately reflects the strategy adopted by the *Carya* Crop Germplasm Committee at their first meeting in 1984: to represent not only grafted accessions representing the pecan industry and named cultivars of hickory, but to represent native populations of all *Carya* species by growing seedlings on their own roots, grown from seed collected from broadly distributed indigenous populations. As a result, the current collection includes an ex situ assemblage of cultivars and wild relatives that represent this important native North American nut crop.

Information related to a living accession in a germplasm collection falls into several interconnected categories: passport descriptors; management descriptors; environment and site descriptors; characterization descriptors; and evaluation descriptors (Alercia et al. 2012; Thormann et al. 2013). The database of the US National Plant Germplasm System, known as the Germplasm Resources Information Network (GRIN-Global), organizes data as recommended by FAO/Biodiversity Multi-Crop Passport Descriptors (MCPD V.2) (Alercia et al. 2012). Whether the accession originated by collection from the wild, or was obtained from another source, critical information is necessary to associate an accession with its history.

Historical records of the Brownwood orchards dating from the early 1930s are maintained as paper files and are consulted for questions of identity or origin. Trees are maintained even when little information is available concerning them. When necessary, accessions have been transferred by grafting to new inventories when original trees are damaged or removed. By associating records of graftwood origin with previous inventories, the line of descent can be traced from the present collection, through the valid evaluation records obtained from previous inventories, back to the ortet, or founding source of the cultivar. By knowing where that original tree grew, the ex situ collection is connected back to the forest, and to a long history of climatic and edaphic adaptation.

Some of the accessions in the initial collections of the NCGR *Carya* had no information other than the name. The purpose of this paper is to describe such an accession phenotypically and genetically, to associate it with the historic cultivar name it is believed to represent, and to describe preliminary efforts to utilize this cultivar in crosses with pecan to produce segregating progeny of utility in the USDA ARS Pecan Breeding Program.

## Methods

The USDA ARS Pecan Breeding and Genetics program maintains two locations where collections of the NCGR *Carya* are held: the original home of the Pecan Breeding Program in Brownwood, Texas; and land in Burleson County, Texas made available through cooperation with Texas A&M University in College Station, Texas.

Molecular profiles have been developed for many accessions in the collection using 14 nuclear microsatellite markers and 3 plastid markers (Grauke et al. 2003, 2010, 2015). Immature leaflets are harvested from specific inventories and frozen at −80 °C until DNA extraction. Molecular profiles were also obtained from samples taken from in situ populations, but not maintained in ex situ collections. Particularly valuable samples were provided by Dr. Donald Stone, Duke University, from collections he made in Mexico in 2001. Those samples were stored at room temperature in silica gel desiccant and sample numbers correspond to the voucher specimens maintained at Duke Herbarium.

Total genomic DNA was extracted as reported in Grauke et al. (2010). Frozen tissue was ground in extraction buffer [0.35 M glucose, 0.1 M Tris–HCl pH = 8, 0.005 M Na_2_EDTA pH = 8, 2% (w/v) polyvinylpyrolidone (PVP-40)] at pH = 7.5 and a lysis buffer [0.1 M Tris–HCl pH = 8, 1.4 M NaCl, 0.02 M Na_2_EDTA pH = 8, 2% (w/v) CTAB, and 2% PVP-40]. During DNA extraction, 1% (w/v) of ascorbic acid and 0.2% (v/v) of β-mercaptoethanol was added to both buffers. DNA was cleaned with chloroform:isoamyl alcohol (24:1) and precipitated with salt and isopropanol or ethanol. PCR reactions were performed as reported in Grauke et al. (2015). PCR fragments were labeled with a fluorescent forward primer at the 5′-end using either 6-FAM (blue color) or HEX (green color). To load the samples on the ABI Prism Genetic Analyzer 3130 (Applied Biosystems, Foster City, Calif. USA), 0.5 μl of the PCR was mixed with 5 μl of 400-ROX internal size standard in deionized formamide at 2.5%. The relative size of the allele was determined using GeneScan and Genotyper software v 3.7 (Applied Biosystems). Alleles were called as a whole number in bp after a binning process with the FlexibinV2 software (Amos et al. 2007). Interpretation of molecular profiles is by visual analysis of allelic patterns within species and hybrids, by geographic regions.

Pollen of ‘Jones Hybrid’ BRW 9-7 was collected on 15, 16 April 2015 for use in making controlled crosses using standard procedures of the breeding program (Grauke and Thompson 1996). Pollen was applied to 80 bagged clusters of 88-FLA-FL-1.1 (*Carya floridana*) on 21 April 2015, and to 170 bagged clusters of ‘Mandan’ pecan on 23 April, 2015. Pollen was stained with 1.5% acetocarmine and photographed under 10X magnification on 28 April 2015.

Pistillate flowers on both Brownwood inventories of ‘Jones Hybrid’ were bagged on 16 April 2015 and pollen of ‘Mandan’ pecan was applied on 23 April 2015. Number of nuts set on all controlled crosses was monitored through the season, and nuts that were set were harvested on 17 September 2015.

## Results and discussion

Historical records include individual tree cards showing the date a seedling was planted, and providing information concerning seedling source, grafting dates, scion identities and sources. Information concerning origin of the scion was not provided for ‘Jones Hybrid’ and no other reference has been found to a hickory tree of this name. The tree labeled as ‘Jones Hybrid’ was established in USDA ARS collections by grafting in 1975. The scion was grafted onto an interstock of B53-16-15 (a ‘McCallister’ seedling) which had been grafted in 1966 onto a pecan seedling rootstock growing in the USDA Brownwood Orchard (BRW) at Row 148, tree 17. ‘McCallister’ is an interspecific hybrid between *Carya laciniosa* and pecan, a hybrid family known as *Carya* × *nussbaumeri*. Records do not indicate a specific cultivar source of pollen for the B53-16 cross series. Open pollinated seedlings would most likely be back-crosses to pecan. The seedling rootstock onto which B53-16-15 was grafted was a controlled cross seedling of the 56-23 series (the 23rd combination of selected pecan parents made in 1956), which had been planted at that location in 1960. The progeny 56-23 was made using pollen of ‘Cherokee’ (‘Schley’ × ‘Evers’) to pollinate flowers of 1944-15-0030 (‘Odom’ × ‘Evers’). The compound tree at BRW 148-17 was removed in 1991, and graftwood of ‘Jones Hybrid’ was collected and grafted to open-pollinated ‘Apache’ seedling rootstocks growing in the newly established Brownwood Variety Orchard (BWV) 5-31 and BWV 9-7. Scions of BWV 5-31 were used to establish a tree at the College Station site, with grafts made to an ‘Apache’ seedling rootstock at CSV 15-20 in 2006.

**Fig. 1:**
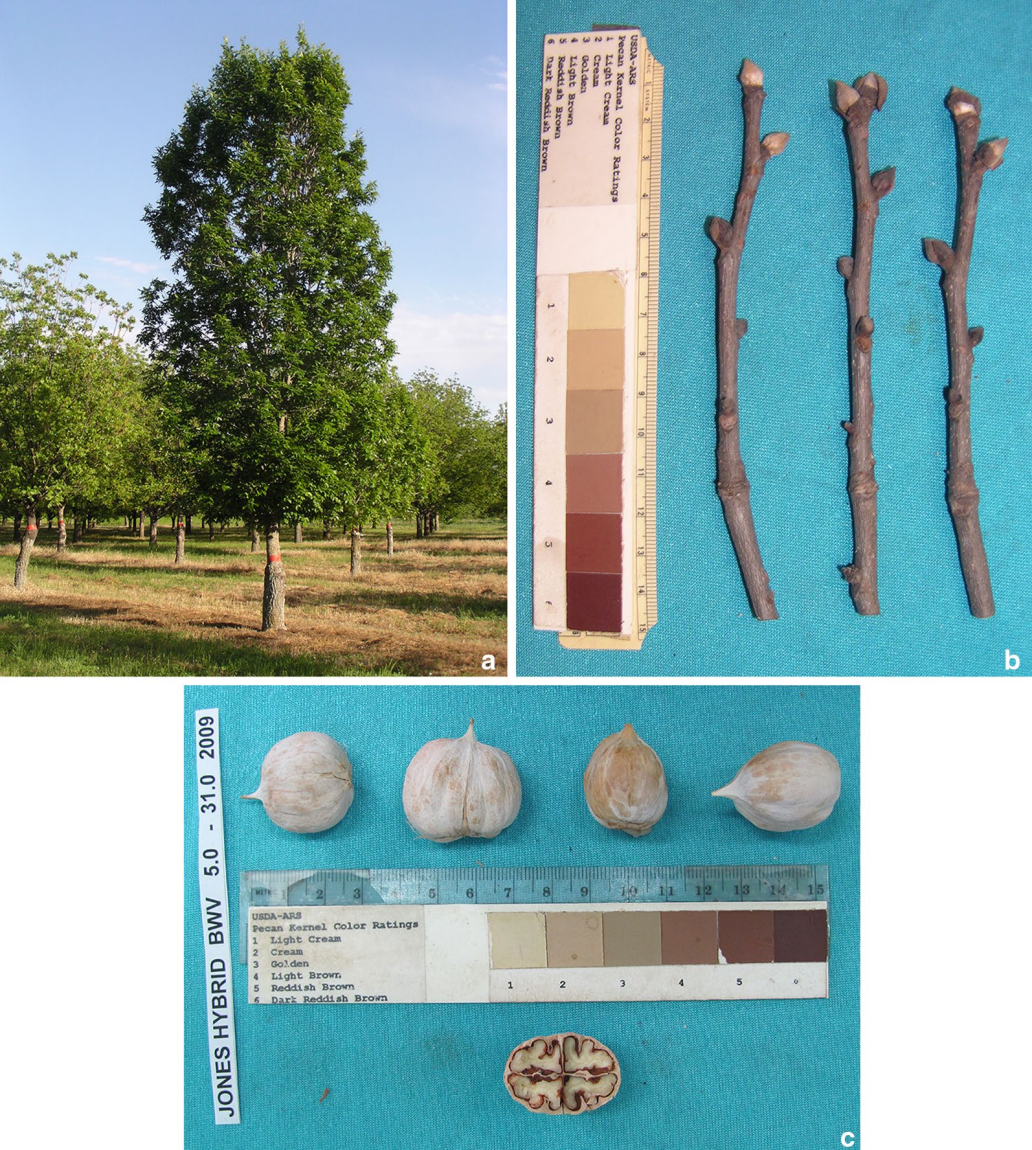
‘Jones Hybrid’ inventory BWV 5-31 of the National Collection of Genetic Resources for Pecans and Hickories; **a** Tree form on 17 Apr 2012 showing columnar habit; **b** bud structure on 3 Mar 2015; **c** nut shape, 2009 crop

Trees of ‘Jones Hybrid’ are noteworthy for their columnar growth habit (Fig. 1a). Dormant winter buds are plump, with imbricate scales (Fig. 1b). Outer bud scales shed in a pattern similar to *C. tomentosa*, but terminal buds are smaller, to 1 cm only. Bark is tight and gray, as is typical of both *C. cordiformis* and *C. tomentosa* and young trees of *C. ovata* prior to bark exfoliation (Grauke 2003).

**Fig. 2:**
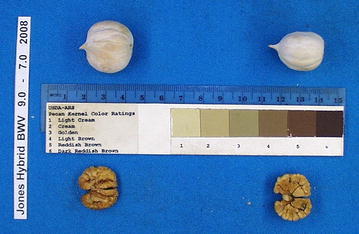
Orbicular nuts of ‘Jones Hybrid’, showing apical stipe and ruminated kernels with prominent basal cleft, traits reminiscent of *C. cordiformis*

**Fig. 3:**
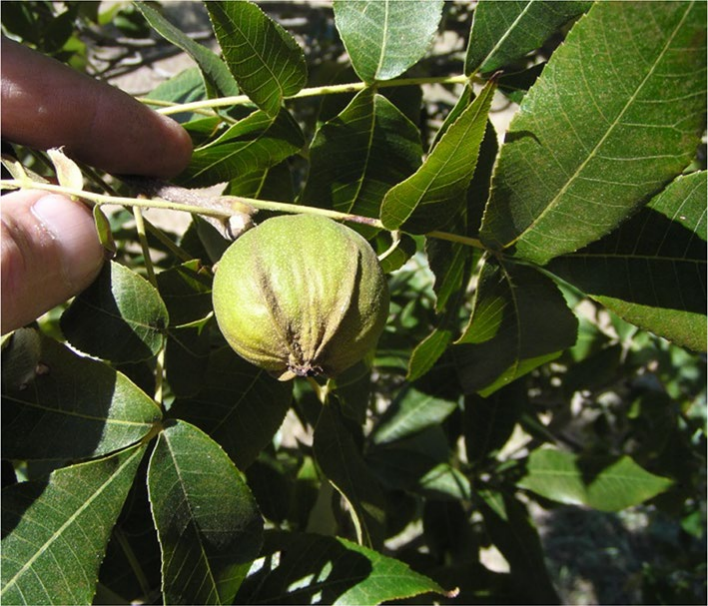
‘Jones Hybrid’ inventory BWV 9-7 fruit, showing wings on shuck sutures from apex to mid-nut. 7 Sep 2011

First fruit was obtained from BWV 9-7 in 2001, the eleventh leaf after grafting, and from BWV 5-31 in 2008 (18th leaf). The involucre or ‘shuck’ of the fruit separates at sutures, or ‘splits’, in late October or early November. Nut samples have been collected, measured and photographed over many seasons (Table 1; Figs. 1c, 2). Nuts are orbicular with a length to height ratio of 1.1; have a prominent apical stipe, and are laterally compressed with a height to width ratio of 1.4 (Grauke and Thompson 2001a, b). Kernels are ruminated with a prominent basal cleft formed by the high secondary septum of the nut, similar to nuts of *C. cordiformis* (Fig. 2). Nuts are borne in shucks with wings on the sutures from the apex to the middle of the nut, typical of *C. cordiformis* (Fig. 3). Leaves are dark green and leathery, with 5-7 oblanceolate leaflets like those of *C. ovata*, but lacking the tufted hairs at the tips of leaf serrations typical of that species. Leaflets are densely pubescent on the lower surface with simple hairs. This trait might suggest a relationship with *C. tomentosa*, although the hairs are mostly single and not the tufted hairs more typical of that species. Leaves are also characterized by short petioles (Fig. 3).

**Table 1:** Nut dimensions of ‘Jones Hybrid’, by inventory and year. Each value is the mean of 5 nuts (except for BWV 9 7 in 2001 and 2009, which had 2 and 4 nuts respectively)

Orchard	Row	Tree	Year	Lng mm	Wd mm	Ht mm	Lng:ht	ht:wd	Nut g	Ker g	Ker pct
BWV	9	7	2001	35	21	30	1.2	1.4	9.3	4.1	44.8
BWV	9	7	2002	33	21	29	1.1	1.4	8.4	4.4	52.4
BWV	9	7	2003	30	20	29	1.1	1.4	8.3	4.1	49.5
BWV	9	7	2005	32	20	28	1.2	1.4	7.6	3.2	42.0
BWV	5	31	2008	30	21	29	1.1	1.4	8.3	4.0	48.6
BWV	9	7	2008	27	19	26	1.0	1.4	6.5	3.3	51.0
BWV	5	31	2009	31	21	30	1.0	1.4	8.4	4.2	49.6
BWV	9	7	2009	30	21	28	1.1	1.4	7.6		
	Mean			31	20	28	1.1	1.4	7.9	3.9	48.6

*Lng* nut length, *Wd* nut width, *Ht* nut height, *Lng:ht* nut length to height ratio, *ht:wd* nut height to width rato, *Nut g* individual nut mass in grams, *Ker g* individual nut kernel mass in grams, *Ker pct* percentage of nut comprised of kernel, *Mean* mean of values across years of measurement

Molecular profiles from the CSV 15-20 inventory of ‘Jones Hybrid’ are shown (Table 2) with those of representatives of other species. The profile of ‘Jones Hybrid’ is typical of those from diploid species: diploid species of *Carya* typically have only two alleles at each nuclear locus, while tetraploids often have as many as four. All profiles observed for the tetraploid species *C. tomentosa*, *C. texana*, *C. floridana* and *C. glabra* have multiple loci with greater than two alleles (all data not shown, but see Table 2, profiles of Scarit *C. tomentosa* and Scarit *C. texana*). The type specimen of *C.* × *collina* (Laughlin 1968)(an interspecific hybrid of those two tetraploid species collected from the Scarit Point, MO population and obtained from Don Stone) has only two alleles at each locus, showing no evidence of polyploidy. Some accessions of *C. ovata* (e.g. ‘Grainger’ and Stone 4160) have more than two alleles at locus *cin4*.

**Table 2:** Nuclear microsatellite alleles in base pairs, alleles arranged vertically by locus for *Carya* species from diverse geographic regions, presented by species within Section and hybrid group

Sample	Section	Spp	State	Accession	*cin22*	*cin13*	*cin20*	*cin4*	*cin23*	*ca10*	*ga38*	*ga39*	*ga41*	*wga242*	*wga118*	*wga321*	*wga4*	*A05*
LJ06-49	apo	cor	LA	02-COR-LA-BF-1	117	126	141	99	83	133	111	112	122	207	177	221	248	110
					117	126	141	99	83	139	111	126	132	211	223	221	270	115
LJ06-50	apo	cor	LA	02-COR-LA-BF-2	117	126	141	90	74	133	111	110	100	209	165	223	258	115
					117	126	141	138	74	139	111	132	100	211	181	223	268	120
LJ06-181	apo	cor	NY	06-COR-NY-1	117	126	141	105	74	139	111	–	106	207	181	221	254	110
					117	126	141	138	83	139	111	–	120	211	193	221	254	115
LJ98-33	apo	cor	KS	90-COR-KS-2	117	126	141	99	74	133	111	112	90	209	173	221	256	105
					117	126	141	99	74	133	111	112	108	223	183	223	260	105
LJ09-903	apo	cor	NY	Schreiner/Stone COR	117	–	141	105	74	139	111	110	90	209	177	221	268	115
					117	–	141	105	83	139	111	110	94	217	177	221	274	115
LJ06-18	apo	ill	TX	Western	114	120	138	93	83	115	117	86	104	213	171	235	242	160
					114	135	138	108	83	115	123	86	104	219	189	259	244	165
LJ06-62	apo/apo	xbr	IL	Abbott Thinshell	114	132	138	93	83	115	109	82	106	207	173	211	242	115
					117	132	141	99	89	133	111	82	106	219	185	257	270	160
LJ06-105	apo/apo	xbr	OK	Pleas	114	132	138	114	83	115	111	96	104	203	175	221	244	115
					117	132	141	144	83	139	129	96	118	209	191	237	244	160
LJ10-4160	apo/car	xio	MX	Stone 4160	108	132	138	93	80	85	111	86	94	217	177	247	228	105
					114	132	138	108	80	115	115	96	104	233	179	247	240	160
								114										
LJ06-55	apo/car	xio	TX	89-XIO-RDM-1	108	120	138	108	80	85	111	86	92	205	175	225	230	105
					114	120	138	108	83	115	117	94	104	219	189	259	242	160
LJ06-115	apo/car	xla	KY	Jones Hybrid	108	132	138	108	80	85	111	98	94	217	175	219	234	105
					108	132	138	117	80	115	111	100	100	243	191	219	240	105
LJ06-180	apo/car	xla	NY	C– × laneyi type	108	120	138	99	89	85	111	108	120	207	169	223	238	110
					117	126	138	138	89	139	111	114	120	209	169	225	280	110
LJ06-42	apo/car	xnu	IN	McCallister	114	132	138	102	80	85	111	88	104	203	165	233	228	105
					114	132	138	102	83	115	115	104	114	231	177	239	250	160
LJ06-114	apo/car	xnu	MO	Wilson	114	129	138	105	80	85	111	104	106	227	169	229	228	105
					114	129	138	108	83	115	111	110	114	231	175	255	242	150
LJ01-453	car	lac	KY	Stevens (LAC)	114	132	138	105	83	85	111	94	94	227	171	229	226	100
					114	132	141	120	83	85	115	104	102	245	177	235	228	110
LJ98-36	car	lac	IN	Stephens(LAC)	114	123	138	96	80	85	111	96	94	227	171	229	226	100
					114	129	141	99	80	115	111	104	102	245	177	235	228	105
LJ06-43	car	lac	–	Nieman	108	132	138	93	80	85	111	102	92	213	179	229	228	105
					114	132	144	93	80	85	111	104	94	215	179	233	228	110
LJ06-44	car	lac	IL	Lindauer	114	132	138	93	80	85	109	94	92	225	183	229	228	105
					114	132	138	93	80	115	111	94	102	225	191	233	228	105
LJ09-905	car	ovt	NY	Schreiner/Stone OVT	108	129	138	114	80	85	111	94	90	233	163	219	226	100
					108	129	138	120	80	85	111	96	94	235	173	251	226	105
LJ10-103	car	ovt	TX	10OVT-6	108	129	138	123	80	85	111	94	94	225	163	219	226	105
					111	129	138	123	80	117	111	94	94	225	175	229	226	105
LJ10-104	car	ovt	TX	10OVT-W1	108	129	138	123	80	85	111	94	94	225	163	219	226	105
					111	129	138	123	80	117	111	94	94	225	175	229	226	105
LJ06-89	car	ovt	LA	01-OVT-LA-2–1	108	129	138	108	80	85	111	94	94	213	173	219	226	105
					108	129	138	117	80	139	111	96	124	213	175	221	234	110
LJ98-38	car	ovt	LA	91-OVT-LA-1	108	129	138	93	80	85	111	94	92	225	163	219	230	105
					108	132	138	120	80	115	111	96	92	225	183	239	230	105
LJ06-67	car	ovt	PA	Seas	108	120	138	93	80	85	111	96	94	221	175	219	226	105
					114	120	138	93	80	85	111	104	94	221	179	235	234	110
LJ10-4131	car	ovt	MX	Stone 4131	108	129	138	99	80	85	111	94	94	223	169	225	228	105
					108	129	138	114	80	85	111	96	134	225	187	233	228	110
LJ10-4161	car	ovt	MX	Stone 4161	108	126	138	114	80	85	111	96	94	217	169	225	226	105
					111	132	138	123	80	139	111	96	94	219	173	241	228	105
LJ06-102	car	ovt	TN	Grainger	108	126	138	105	80	85	109	94	94	209	183	225	226	105
					114	129	138	114	80	85	111	102	106	227	191	231	244	105
								117										
								120										
LJ06-113	car	ovt	OH	Yoder 1	108	120	138	108	80	115	109	102	94	229	171	235	226	105
					114	132	138	108	80	115	111	102	94	229	185	245	234	105
LJ06-100	car	ovt	IL	01-OVT-IL-8–3	108	129	138	117	80	85	109	100	96	219	163	223	228	105
					114	129	138	117	80	85	111	100	106	225	177	223	228	105
LJ09-1816	car	tom	MO	Scarit 1816 TOM	108	120	138	108	80	85	111	104	94	–	169	225	238	110
					114	132	141	120	89	115	111	110	104	–	179	233	240	115
								126								237	244	120
																253		
LJ09-1814	car	tex	MO	Scarit 1814 TEX	108	–	141	98	80	85	107	100	94	–	167	225	226	110
					108	–	144	108	80	115	111	100	104	–	169	233	254	115
								129			113				171	237		
															179			
LJ09-828	car	flo	FL	09FLA-ABS1	108	120	138	99	77	115	111	98	94	217	171	225	234	–
					108	132	141	99	80	139	111	102	112	223	181	227	238	–
						138	144							227	185		248	
														233			290	
LJ09-1812	car/car	xco	MO	Scarit 1812 XCO	108	120	138	102	77	85	111	–	90	–	175	–	226	105
					114	132	138	114	80	115	119	–	104	–	175	–	228	115

Profiles for Stone 4131, 4160 and 4161, are matched by herbarium vouchers with those numbers in the Duke Herbarium

Profiles for Scarit 1812, 1814 and 1816 are matched by herbarium vouchers with those numbers in the Duke Herbarium

Section *Apocarya* = apo; Section *Carya* = car; hybrids by Sections = apo/apo, apo/car, or car/car; Species: *C. aquatica* = aqu; *C. cordiformis* = cor; *C. illinoinensis* = ill, *C. floridana* = flo; *C. laciniosa* = lac; *C. ovata* = ovt; *C. texana* = tex; *C. tomentosa* = tom; *C.* × *brownii* = xbr (ill × cor); *C.* × *collina* = xco (tom × tex), *C. illinoinensis* × *C. ovata* = xio, *C.* × *nussbaumeri* = xnu (lac × ill), *C.* × *laneyi* = xla (cor × ovt)

All accessions of *C. cordiformis* examined have the 117 bp allele at the *cin22* locus and the 141 bp allele at the *cin20* locus, while previously examined interspecific hybrids have those two alleles plus a second allele from the other parent (Table 2). Note that the profile from the type tree of *C.* × *laneyi* (*C. cordiformis* × *C. ovata*)(LJ06-180) from Riverside Cemetery in Rochester NY has the 117 bp allele from *C. cordiformis* (Table 2). None of the typical *C. cordiformis* alleles are found in ‘Jones Hybrid’. However, there is clear morphological evidence of hybridity with *C. cordiformis* in the nut, kernel and shuck characteristics. The 108 bp allele of *cin22* has been found in all *C. ovata* examined and is present in ‘Jones Hybrid’, which is consistent with parentage by that species. However, that allele is also found commonly in the tetraploids *C. texana* and *C. floridana* and in some accessions of *C. tomentosa* (Table 2).

Chloroplasts are maternally inherited, and allele sizes at *ccmp2*, *ntcp40* and *ntcp9* are presented in Grauke et al. (2010). ‘Jones Hybrid’ has alleles sizes 209, 196 and 351 bp at these three loci, which is a haplotype that has not been observed in *C. cordiformis*. That pattern is among the most common plastid haplotypes found in *Carya*, and has been observed in *C. aquatica*, *C. illinoinensis*, *C. palmeri*, *C. glabra*, *C. myristiciformis*, *C. laciniosa*, *C. ovata*, *C. texana*, and *C. tomentosa* (Grauke et al. 2010). That haplotype has not been observed in *C. cordiformis*. If ‘Jones Hybrid’ is an interspecific hybrid between *C. ovata* and *C. cordiformis*, this suggests that the maternal parent was *C. ovata*. By contrast, the plastid haplotype of the *C.* × *laneyi* type tree (LJ06-180) in Rochester (208/197/350 bp) is shared by a local *C. cordiformis* (06-COR-NY-1, Sample LJ06-181) (data not shown).

‘Jones Hybrid’ pollen dehisced prior to pistillate receptivity, inferring that the accession is protandrous. No nuts were set on trees of ‘Jones Hybrid’ pollinated using ‘Mandan’ pollen. No nuts were set on the tetraploid *Carya floridana* tree pollinated using ‘Jones Hybrid’ pollen. Of 33 nuts initially set on ‘Mandan’ trees pollinated using ‘Jones Hybrid’ pollen, only three remained at harvest.

**Fig. 4:**
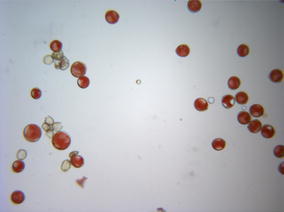
Pollen from ‘Jones Hybrid’ BWV 9-7, collected 15 Apr 2015, stained with acetocarmine 28 Apr 2015, imaged using a ×10 objective lens, showing diversity of pollen size and viability, including the presence of “micro” (tiniest) and small unstained pollen, and a wide range of sizes among stained pollen grains

Pollen grains from ‘Jones Hybrid’ BWV 9-7 were variable in stain absorption and size (Fig. 4). Grains lacking stain are inferably non-viable and nonfunctional, whereas stainable ones would potentially be viable and functional. Strong variation in size suggests variation or inconsistency in numerical patterns of meiotic chromosome disjunction. Presence of “micro” pollen suggests formation of meiotic products with just one or a few chromosomes, also an indication of perturbed meiosis. Among stained grains, the range in diameters was wide enough (ca. 1.29-fold) to possibly indicate presence of haploid (n), diploid (2n) pollen and/or related aneuploid grains, where 2n grains would be unreduced (diploid) and potentially fertile, along with haploid grains (Fig. 4).

**Fig. 5:**
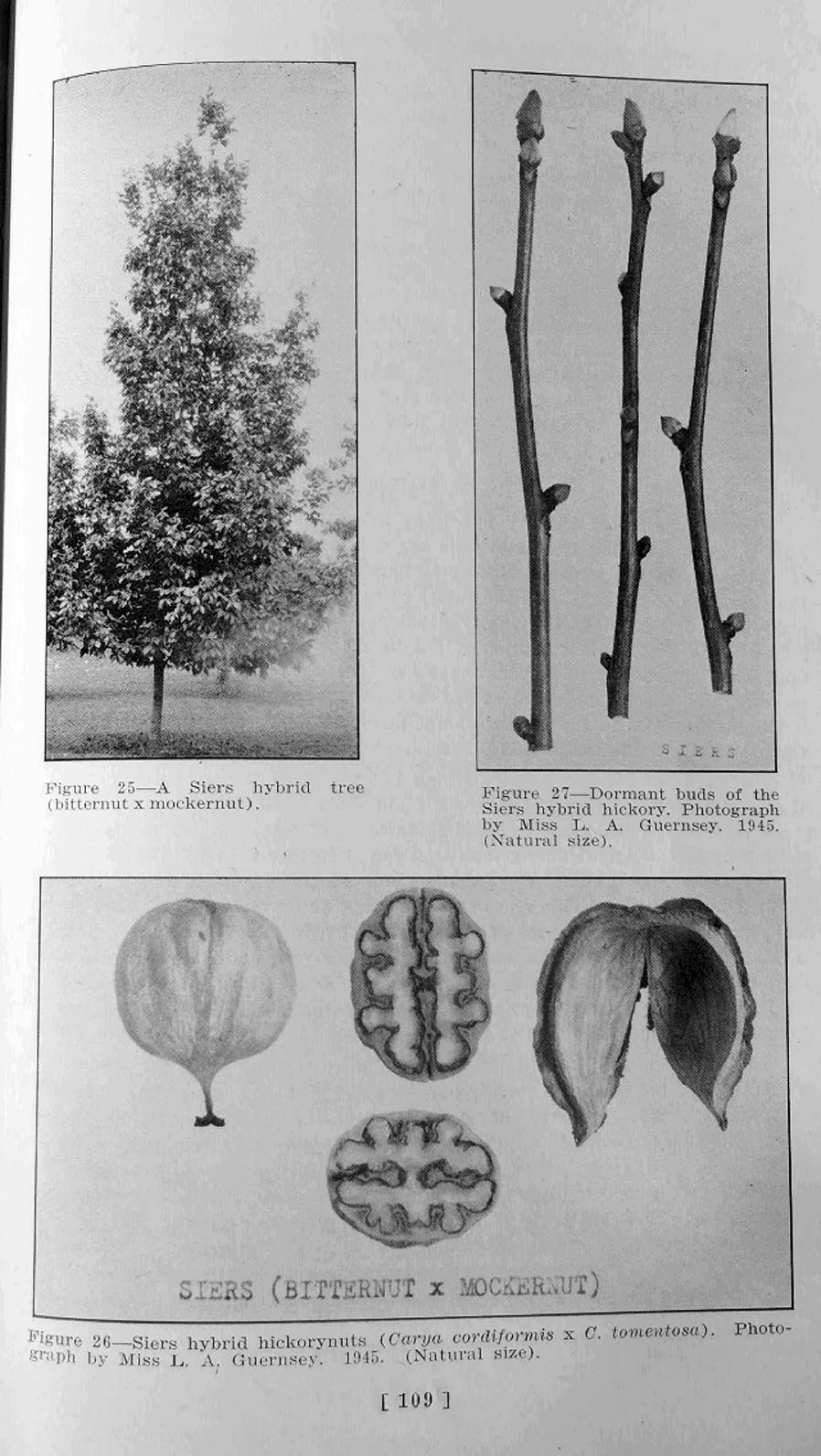
Reproduction of Figs. 25, 26, and 27 from Reed (1944) showing pictures of the columnar tree form, plump buds with shed outer bud scales, and characteristic apiculate, laterally compressed nuts with shucks

The association of morphological traits linking *C. cordiformis*, *C. tomentosa* and *C. ovata* suggested comparison of the ‘Jones Hybrid’ with the ‘Siers’ hybrid, reported to be a cross between *C. cordiformis* and *C. tomentosa* in some references and *C. ovata* × *C. cordiformis* in others (Grauke 1988). The oldest reference to ‘Siers’ described it as “a hybrid pecan” entered in a nut contest conducted by the Northern Nut Growers Association in 1915 (Deming 1929). ‘Siers’ was thoroughly described by Reed (1944) who provided pictures of the tree, buds, nuts and shucks (Fig. 5). Comparison of Reed’s (1944) figures (Fig. 5) to Fig. 1 in this report shows striking similarity. Cross sections of nuts in Reed’s (1944) figure have nut height to width ratios averaging 1.36, close to the value (1.39) for ‘Jones Hybrid’ (Table 1). Reed (1944) noted that the tree had “dark and rather rough” leaves, and “the petiole below the lowermost leaflets is not more than 3 inches in length.” Kernels were described as deeply divided, pink to reddish, and often astringent. All descriptions are consistent with current and previous observations on ‘Jones Hybrid.’

The original ‘Siers’ tree was found around 1905 by Dr. I. T. Siers of Lawrenceburg, KY, who estimated its age in 1916 to be 25 years (germinating around 1891). Trees were first propagated in 1916 by the J. F. Jones Nursery, Lancaster, PA. Reed (1944) noted that “propagation by nurserymen appears to have ceased by 1930”, due to kernel astringency. Wyman (1942) reported that trees of ‘Siers’ (*C. cordiformis* × *C. tomentosa*) were offered for sale by E. A. Riehl Nursery of Godfrey, IL, but not by J. F. Jones Nursery who had other pecan, hickory and hybrid cultivars listed. Among the listed hazelnut and filbert cultivars being sold by J. F. Jones Nursery was one named “Jones Hybrid.”

‘Siers’ was listed as a “shagbark-bitternut” (=*C. ovata* × *C. cordiformis*) hybrid by L. H. MacDaniels (1969, 1979), describing it in both places as a “tall slender ornamental; low quality nut. Formerly, and probably erroneously, called a mockernut-bitternut hybrid” [*sic*] (=*C. tomentosa* × *C. cordiformis*).

Evidence indicates that ‘Jones Hybrid’ in our collection is ‘Siers.’ Adding ‘Siers’ as a synonym to the record will associate the history of that cultivar with our accession, and provide a geographic point of origin for evaluation in further testing. It is more interesting that several other prominent interspecific hickory hybrids originate in that geographic region: ‘McCallister,’ among the most prominent cultivars of *C.* × *nussbaumeri*, originated just north of the Ohio River near Mount Vernon, IN; ‘Major,’ a pecan that has contributed to several cultivars released by the USDA ARS Pecan Breeding Program, originated in the Henderson, KY area on the Green River and carries alleles from *C. cordiformis* and possibly *C. ovata* (Grauke et al. 2015). Hybridity occurs in areas of sympatry, and that region has the highest concentration of sympatric *Carya* species (Grauke and Mendoza-Herrera 2012).

## Conclusion

Until more convincing evidence is found linking the accession to *C. tomentosa*, the ‘Jones Hybrid’=’Siers’ will be considered a hybrid between *C. ovata* and *C. cordiformis*, the *C.* × *laneyi* family of hybrids. The facile hybridizations of *C. ovata* with other diploid *Carya* species, the genetic anomalies seen in pollen of this hybrid accession, and allelic associations between *C. ovata* and the tetraploid species suggest not only that *C. ovata* may be a progenitor of the tetraploid species of *Carya*, but that its hybrids may provide a bridge for valuable traits of tree shape and size control.

Preliminary efforts in constructing controlled cross progenies were unsuccessful. Whereas ‘Jones Hybrid’ pollen applied to diploid pecan led to a very limited amount of fruit, indicating fertility of haploid grains, pollen applied to tetraploid *C. floridana* did not. The pollination results would seem to suggest that the larger pollen grains were not diploid, or if so, that they were non-functional. Other possible explanations exist, however, e.g., the pistillate flowers may have been past receptivity (control pollinations were not made at the same time), or other issues precluded function. Additional efforts will be needed to fully determine if 2n pollen are produced and are functional.

The wild relatives of pecan provide abundant diversity for the future development of this valuable tree crop. Their exploitation will require improved genomic tools to facilitate strategic phenotypic selection. The tall, slender tree form of ‘Jones Hybrid’ could be valuable in commercial pecan cultivars, allowing increased tree densities and reducing the need for expensive hedging operations. Tree size reduction is a goal to be pursued not only in scion selection, but through rootstock development. Negative traits such as long juvenility, low productivity, poor kernel percentage and astringent kernel flavor all need to be avoided as positive aspects of columnar tree form and reduced tree size are pursued. Development of molecular genetic markers associated with traits will assist selection. Strategic use of the accessible diversity in collections of the NCGR *Carya* will provide the foundation for development of those tools for pecan, the most important native North American nut crop.
